# Some Experiences of Training School Life

**Published:** 1918-08-10

**Authors:** 


### THE MATRONS' AND SISTERS' DEPARTMENT.

### SOME EXPERIENCES OF TRAINING-SCHOOL LIFE.

### III. Are the Hardening Influences Remediable?

The hardening influences of hospital life result to such a large extent from the very forces which must of necessity be exercised to produce a highly trained, skilled and capable nurse, that the question is one involving many complications and subtleties. The most that can be done on paper is to indicate the directions whence remedies might come:

The difficulty of over-strain from lack of time is not necessarily removed by half-empty wards and very little work, for the ideal of a good nurse is

☞ Previous articles appeared July 20, and August 3, p. 393.

**Some Experiences of Training-School Life**—(*cont.*)

plenty of work to do and sufficient time to do it properly. If she is fit and well the methodical nurse—whether she be slow or quick by nature—will reach the necessary standard of speed in accomplishing her work without losing anything of her natural gentleness and consideration. Shorter hours and longer holidays are essential therefore, not only as part of a nurse's Charter of Liberty, but from every point of view.

The force of example should in every hospital be an influence for good instead of evil. The senior nurses do more to set the tone than perhaps any other section, and upon their realisation of their responsibilities the probationers' future largely depends; yet too frequently they show an extraordinary facility in forgetting what the hospital world looks and feels like to the "pro." They have wonderful opportunities—far more than the matron, more even than the sisters, for these are sometimes regarded as a race apart. At the same time they have their own special difficulties to face, and are often taken up with what may be called their newly-acquired technical responsibilities. Could not something be done to make them realise their deeper responsibilities as the immediate examples of their juniors?

The hardness that results from familiarity is an influence not easily eliminated. As already pointed out, it is often an entirely unconscious development. The writer recalls an instance in her own experience, of an extremely good nurse who developed such an extraordinary roughness in removing the dressings from the soldiers' wounds that they dreaded her appearing. By some accident this came to her knowledge. So unconscious had the development been that it was a genuine shock to her to learn the truth, and as a result, not only did she inflict upon herself the penalty of refusing to wear any more an honour previously won, but never again was a single murmur heard in her ward, and the soldiers learned to love her for her gentleness. Surely the lecture on the ethics of nursing which comes in some form into the training of nearly every nurse should deal with this danger, and be followed up in some way without giving the nurse the impression that she is being "preached at." No antidote can be provided, for that lies wholly in the spiritual and mental development and outlook of the nurse herself, but at least the stumbling-block can be pointed out as something to be avoided.

An exclusive absorption in the "case" often becomes a real danger to nurses. The interest of theoretical study and the excitement of examinations heighten the danger, and yet at the same time it must be realised that this intellectual interest is not only a valuable asset from the technical point of view, but it is in many cases a most useful check upon an over-developed human interest—which in the absence of wisdom and balance on the part of the nurse may constitute a danger in the opposite direction. This is one of those subtle complications which no law can unravel, and the golden mean is not easy of attainment. Here again, however, a note of warning wisely given early in training will put the nurse on her guard against the hardening influence.

The natural reaction against the helplessness of mere words, leading to an exaggerated assumption of indifference which in time becomes hardness, is incapable of remedy from without. To a certain extent it is inevitable and almost desirable: for the nurse whose sympathy and sensitiveness interfere with her skill, good judgment, or ability to deal firmly with her patients is not attaining the highest of which the profession is capable. The remedy is a personal matter of character. A very gentle hint from her matron, or from a sister old enough and wise enough to read clearly the underlying character and see the difficulty, may lead to confidence and understanding, and be of real help to the nurse in solving her own problem.

The development of hardness is thus seen to be very largely a question of personal character, which must be dealt with individually, and with great wisdom and tact. At the same time, much could be done during training. It is not the occasional sermons that bring these things home to nurses: these they hear, and either completely ignore, occasionally make fun of, or else soon forget. It is the whole tone and temper of the hospital and the standard that is set; the recognition of the difficulties and dangers of a nurse's life, and the dealing with them, not in a sentimental spirit, nor yet with a repressive schoolmistress attitude, but with a frank and practical appeal to all that is best in the woman who comes to the hospital to be made into "a good nurse."

